# Heavy alcohol intake is a risk factor for esophageal squamous cell carcinoma among middle-aged men: A case-control and simulation study

**DOI:** 10.3892/mco.2013.142

**Published:** 2013-07-04

**Authors:** NAOKO KUMAGAI, TOSHIFUMI WAKAI, KOHEI AKAZAWA, YIWEI LING, SHIJIE WANG, BAOEN SHAN, YOSHIYASU OKUHARA, YUTAKA HATAKEYAMA, HIROMI KATAOKA

**Affiliations:** 1 Center of Medical Information Science, Kochi Medical School, Kochi University, Nankoku, Kochi 783-8505, Japan; 2 Integrated Center for Advanced Medical Technologies, Kochi Medical School, Kochi University, Nankoku, Kochi 783-8505, Japan; 3 Division of Digestive and General Surgery, Niigata University Graduate School of Medical and Dental Sciences, Niigata, Niigata 951-8510, Japan; 4 Department of Medical Informatics, Niigata University Medical and Dental Hospital, Niigata, Niigata 951-8520, Japan; 5 Fourth Affiliated Hospital of Hebei Medical University, Shijiazhuang, Hebei 050011, P.R. China

**Keywords:** alcohol intake, conditional logistic regression analysis, esophageal squamous cell carcinoma, matched case-control study, middle-aged Chinese men, simulation

## Abstract

Despite the advances in surgical techniques and treatments, the prognosis of esophageal cancer remains poor, since the disease is usually diagnosed at an advanced stage. Therefore, prevention plays an important role in reducing mortality. Smoking and alcohol intake are modifiable habits and are important risk factors for esophageal cancer. However, the number of large-scale studies that have investigated the association of the amount and duration of smoking and alcohol intake with esophageal cancer risk, while accounting for the effects of gender and cancer subtypes (squamous cell carcinoma and adenocarcinoma), is limited. Therefore, in this hospital-based matched case-control study we investigated this association while accounting for gender and subtype differences. Chinese male patients <60 years of age with esophageal squamous cell carcinoma (ESCC) from the Fourth Hospital of Hebei Medical University in China and healthy individuals were enrolled between January, 2002 and December, 2006. Each ESCC patient was age-matched to a control subject and a total of 535 pairs were enrolled in this study. The combined variables of amount and duration were created to elucidate their effect and association with ESCC. Multiple conditional logistic regression analysis was used to estimate the odds ratio (OR) and 95% confidence interval (CI) in this model, which included a family history of esophageal cancer, a combined smoking variable and a combined alcohol variable. A simulation study was subsequently performed to confirm the reliability of the results. The results of the present study demonstrated that a family history of esophageal cancer and the combined alcohol variable were significantly associated with ESCC risk. Heavy alcohol consumption and intake for ≤20 years increased the risk compared with no intake (OR=1.91, 95% CI: 1.25–2.92). Heavy alcohol consumption and intake for >20 years exhibited an even higher risk (OR=7.25, 95% CI: 3.12–16.83). These results were similar to those of the simulation. Heavy alcohol intake, even for a short duration, is a critical risk factor and may lead to the development of ESCC in Chinese males.

## Introduction

The pathogenesis of esophageal cancer has not been fully elucidated and, despite the advances in surgical techniques and treatments, its prognosis remains poor, since the disease is usually diagnosed at an advanced stage (1–5). Thus, prevention plays an important role in reducing mortality. Numerous risk factors for esophageal cancer have been identified, including tobacco smoking, alcohol intake, hot beverage intake, family history of cancer and low fruit and vegetable intake (4–8), the first two are considered to be the most significant factors. These habits are correlated with the disease at the individual level and are potentially modifiable (9). Notably, the incidence of esophageal squamous cell carcinoma (ESCC) is on the decrease in the USA in association with a decline in smoking and alcohol intake (10).

Several studies investigating the effects of smoking and alcohol intake on the risk of esophageal cancer have demonstrated that long duration, high consumption and the interaction of these habits may increase the risk of ESCC (9,11–17). However, with the exception of two studies which demonstrated that the effects of smoking and alcohol intake on esophageal cancer may differ according to gender (9,18), to the best of our knowledge, only a limited number of large-scale studies have investigated the association of the amount and duration of smoking and alcohol intake with the risk of esophageal cancer, while accounting for gender-specific effects.

In this study, the incidence of ESCC among Chinese males was investigated by performing conditional logistic regression analysis of the combined effects of the amount and duration of smoking and alcohol intake on ESCC risk in a relatively large study population of 1,070 males from a hospital-based casecontrol study. The combined effects were estimated using a variable that combined amount and duration. The reliability of our results was subsequently confirmed by means of a simulation. The aim of investigating the combined effects of amount and duration of smoking and alcohol intake on the risk of ESCC was to provide sufficient data to guide improvements in prevention strategies.

## Materials and methods

### Design

In this case-control study, each patient was age-matched to a control subject (1:1 ratio) and a total of 535 pairs were enrolled.

### Participants

A total of 571 Chinese male ESCC patients, aged <60 years, were enrolled from the Fourth Hospital of Hebei Medical University in Shijiazhuang, China. They were all residents of Shijiazhuang and between January, 2002 and December, 2006 had been diagnosed with histologically confirmed primary invasive ESCC, as defined by the International Classification of Diseases, 10th revision (ICD-10) diagnostic code (code, C15). In addition, 1,307 healthy male subjects, aged <60 years, were selected from the same area and during the same time to serve as controls. All controls had undergone medical checkups that verified the absence of any medical conditions. The participants were informed on the study objectives and content and provided informed consent prior to enrollment. The study protocol was approved by the local Ethics Committee and the Research Committee of Hebei Medical University.

### Risk factors

Trained interviewers used a structured questionnaire to interview the participants. The questionnaire included questions on demographic factors such as age, gender, smoking behavior (age of initiation and cessation of smoking, duration of smoking and the number of cigarettes per day), alcohol intake (age of initiation and cessation of alcohol intake, duration of alcohol intake and type and the quantity of alcoholic beverages consumed) and the family history of esophageal cancer. A non-drinker was defined as someone who consumed <1 alcoholic drink/month. Daily alcohol intake (g ethanol/day) was calculated by considering the beverage type and daily consumption. Beverage type was classified as *baijiu* (the most popular Chinese alcoholic beverage), beer or other hard liquor. One unit of consumption was defined as 50 ml of *baijiu*, 400 ml of beer or 50 ml of hard liquor. The content of pure alcohol was calculated as follows: 40% for *baijiu*, 5% for beer and 40% for hard liquor. The resulting values were expressed in grams by converting 1 ml of pure ethanol to 0.789 g.

All continuous variables (number of cigarettes/day, duration of smoking, daily alcohol intake and duration of alcohol consumption) were assigned to categories of zero, low and heavy. Absence of smoking or alcohol intake was categorized as zero (reference group). To ensure equal sample sizes, median values were used to divide tobacco smokers and alcohol drinkers into low and heavy groups. Values ranging from >0 to the median were classified as low and values greater than the median were classified as heavy. The duration and amount categories were subsequently combined to investigate the association of duration and amount of smoking or alcohol intake with ESCC risk. The combined smoking variable comprised 5 categories: no smoking, ≤20 cigarettes/day for ≤20 years, ≤20 cigarettes/day for >20 years, >20 cigarettes/day for ≤20 years and >20 cigarettes/day for >20 years. Five combined categories were created for alcohol intake: no alcohol intake, ≤53.3 g ethanol/day for ≤20 years, ≤53.3 g ethanol/day for >20 years, >53.3 g ethanol/day for ≤20 years and >53.3 g ethanol/day for >20 years.

### Statistical analysis

The individual risk factors for ESCC (i.e., family history of esophageal cancer, combined smoking variable and combined alcohol variable) were assessed by univariate conditional logistic regression analysis, and unadjusted ORs and 95% CIs were estimated. Multivariate conditional logistic regression analysis was used to assess the association between the amount and duration of smoking and alcohol intake and the risk of ESCC. Fisher’s exact test was used to compare the proportion of ESCC cases between males aged 30–49 and 50–59 years for each category of the combined alcohol variable. Each proportion was shown in a bar graph to demonstrate the likelihood of developing ESCC at a younger or older age. P<0.05 was considered to indicate a statistically significant difference. P values were derived from two-sided statistical tests. Statistical analysis was performed using SAS statistical software version 9.2 (SAS Institute Inc., Cary, NC, USA).

Although the control for each case was randomly selected from the candidate pool comprising several controls of matching age, the OR estimates may have been inconsistant, depending on the control selected. Therefore, a simulation study was performed to evaluate the reliability of the OR estimates obtained from the multivariate conditional logistic regression analysis. Each simulation dataset included the 535 age-matched pairs. The controls were randomly extracted from the original database of 1,307 male controls, without replacement. For each dataset, conditional logistic regression analysis was performed with the previous three variables. This process was repeated 10,000 times, thus generating 10,000 estimates of coefficients and standard errors for the variables. The simulation generated means for coefficients and standard errors, which were used to produce a summary of ORs and 95% CIs.

## Results

### Participant characteristics

Selected characteristics of patients and controls are presented in Table I. A total of 772 subjects (72.2%) were aged 50–59 years and 270 (25.2%) were aged 40–49 years. These age groups represented 97.4% of the participants. Overall, 82.8% of the alcohol drinkers also had a smoking habit.

### Univariate conditional logistic regression

The univariate conditional logistic regression analysis revealed that family history of esophageal cancer, the combined smoking variable and the combined alcohol variable were significantly associated with ESCC risk (P<0.001) (Table I). With regard to smoking, a duration of ≤20 years was not a significant risk factor, irrespective of the quantity of cigarettes smoked. With regard to alcohol intake, all categories of the combined alcohol variable, apart from an intake of ≤53.3 g ethanol for ≤20 years, were significantly associated with ESCC risk.

### Multivariate conditional logistic regression

Multivariate analysis revealed that a family history of esophageal cancer and the combined alcohol variable were significantly associated with ESCC risk (P<0.001), although the combined smoking variable was not (Table II). The OR (95% CI) associated with a family history of esophageal cancer was 2.06 (1.48–2.85). With regard to the combined alcohol variable, the OR (95% CI) associated with an intake of ≤53.3 g ethanol/day for ≤20 years was 1.20 (0.83–1.74); with an intake of ≤53.3 g ethanol/day for >20 years, 2.28 (1.32–3.94); with an intake of >53.3 g ethanol/day for ≤20 years, 1.91 (1.25–2.92); and with an intake of >53.3 g ethanol/day for >20 years, 7.25 (3.12–16.83). All the categories of the combined alcohol variable, apart from an intake of ≤53.3 g ethanol/day for ≤20 years, were significantly associated with risk. Alcohol intake for >20 years was a risk factor, as was a heavy alcohol intake of >53.3 g ethanol/day. Heavy alcohol intake was significantly associated with ESCC risk, even if the duration of alcohol intake was ≤20 years.

### Comparison of the proportion of ESCC among males aged 30–49 and 50–59 years by category of combined alcohol variable

**Figure 1:**
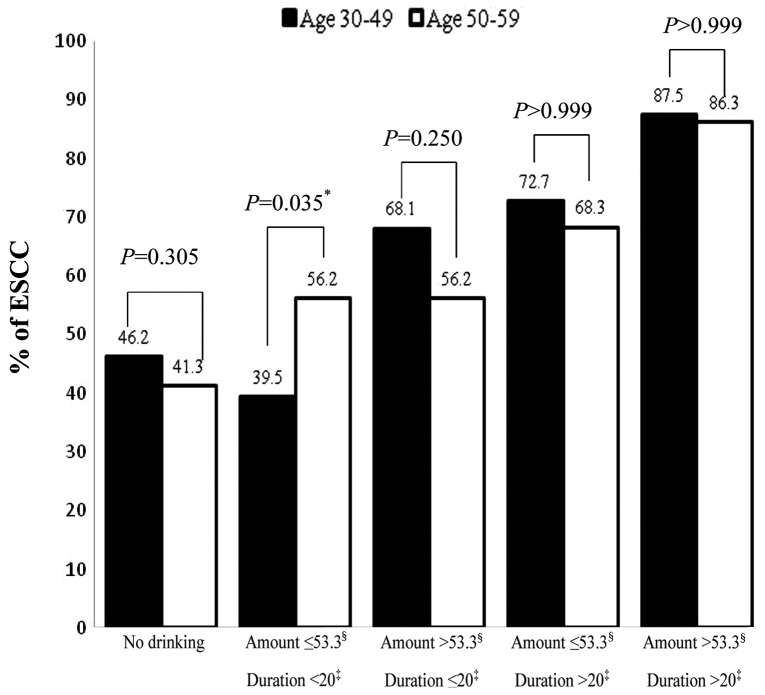
Comparison of proportions of esophageal squamous cell carcinoma (ESCC) in males aged 30–49 and 50–59 years using Fisher’s exact test. ^*^Significant difference between age groups (P<0.05). ^‡^Duration (years) of alcohol intake. ^§^Quantity of alcohol intake (g ethanol/day).

Among males who have consumed alcohol for ≤20 years, the proportion of ESCC cases was higher among older males compared with younger males with an intake of ≤53.3 g ethanol/day (P=0.035) (Fig. 1). By contrast, the proportion was higher among younger males compared with older males with an intake of >53.3 g ethanol/day, although the difference was not statistically significant (P=0.250). Thus, heavy alcohol intake may lead to the development of ESCC at a younger age.

### Simulation

The multivariate conditional logistic regression and corresponding simulation study demonstrated that the same factors were significantly associated with ESCC risk (Table II).

## Discussion

Our analysis indicated that heavy alcohol intake (>53.3 g ethanol/day) is an important risk factor for ESCC in middle-aged Chinese males. In addition, heavy alcohol intake, even of short duration, may lead to the development of ESCC at a younger age. Smoking was not identified as a significant risk factor for ESCC.

We investigated ESCC in a cohort of middle-aged Chinese males, aged <60 years, for the following reasons: although alcohol intake may be a dominant risk factor for both sexes, a previous study by Wu *et al*(18) revealed that alcohol intake increased the risk of ESCC risk in males, with an OR of 1.76 (95% CI: 1.48–2.09). However, the association was not significant in females, a finding which was supported by those of Castellsagué *et al*(9), who observed that alcohol intake was associated with esophageal cancer in males, with an OR of 4.4 (95% CI: 3.1–6.2) and to a lesser extent in females, with an OR of 2.2 (95% CI: 1.3–3.9). Against this background, Chinese males were focused on in this study, as it would appear that they may be at higher risk of developing ESCC compared to females. Moreover, with regard to age, patients and controls aged <60 years were recruited in this study in order to avoid the numerous factors associated with cancer differentiation and development in elderly adults.

Our study demonstrated a stronger association of ESCC development with heavy alcohol intake, compared to long duration of alcohol intake, confirming the findings of previous studies (14,16,17). When daily ethanol consumption exceeded 53.3 g, the risk of developing ESCC within the next 20 years was increased. The exact mechanism by which ethanol causes esophageal cancer has not been elucidated, although several possible pathways have been proposed. Although ethanol itself is not carcinogenic, its major intermediary metabolite, acetaldehyde, is a known carcinogen in animals; alcohol may act as a solvent that enhances the penetration of carcinogens from other environmental sources; the regular intake of alcohol consumption may reduce the intake and bioavailability of certain nutrients that have chemopreventive properties; and alcohol may directly irritate the esophageal epithelium, creating the potential for ESCC pathogenesis (20). Whereas heavy alcohol intake may yield more acetaldehyde and reduce the intake and bioavailability of certain nutrients, the solvent properties of alcohol and its ability to directly irritate the esophageal epithelium are most likely causes for our observations in middle-aged Chinese males. Firstly, the majority (82.8%) of the alcohol drinkers in our study population were also smokers. Tobacco contains the chemical carcinogen nitrosamine, as well as other cancer-promoting agents (5). These agents may act synergistically with ethanol to increase ESCC risk (5) and our results may be explained by the concurrence of the two habits. Secondly, it has previously been demonstrated that exposure to a high concentration of ethanol (40%) severely damaged the esophageal mucosa of rabbits, whereas a lower concentration (20%) exerted a notably lower adverse effect (21). Furthermore, consumption of undiluted hard liquor (>40% ethanol) was found to be significantly harmful, whereas wine, beer and hard liquor with a non-alcoholic mixer were not (11,20). With an ethanol content of 40–60%, *baijiu* is consumed undiluted (22) and is the beverage of choice among Chinese males, accounting for >1/3 of all alcoholic beverages consumed in China (22). The high concentration of alcohol may directly irritate the esophageal epithelium and lead to the development of ESCC within a short period of time, i.e., at a younger age.

Although a previous meta-analysis of worldwide data demonstrated that smoking is an established risk factor for esophageal cancer (23), according to our data it was not a significant risk factor among our cohort of middle-aged Chinese males. Evidence on the ESCC risk associated with smoking in China is inconsistent. Weak associations with tobacco have been reported in Chinese regions exhibiting a significantly high risk for esophageal cancer, such as Linxian, whereas strong associations have been reported in low-risk urban areas (5,6,20,24). The reason for the weaker association in these areas compared to the rest of the world has not yet been elucidated. One hypothesis is that there are other important risk factors in these high-risk areas and that these factors account for the majority of ESCC cases, thus reducing the effect of smoking (6). The lack of association in the present study may also be due to the effect of other risk factors.

This hospital-based case-control study has several limitations. Although the age-matched case-control design was used to decrease the effects of potential confounding factors, ESCC risk was estimated by only using information on alcohol intake, smoking and family history of esophageal cancer. Other categories of smoking and drinking, such as currently or formerly practiced, were not investigated. Moreover, heavy alcohol intake may be correlated with nutritional, socioeconomic or educational status, although we were unable to collect data on other possible risk factors, such as diet and social status. However, we believe that the limitations mentioned above did not significantly affect the study findings, as the differences among groups were too clear to have resulted from such bias.

In conclusion, heavy alcohol intake, even of short duration, is an important risk factor for ESCC in middle-aged Chinese males. Specifically, middle-aged Chinese males who consume >53.3 g ethanol/day (~170 ml of *baijiu*) are at high risk of developing ESCC, even with a duration of heavy alcohol intake of <20 years. This finding indicates that such individuals are at high risk of developing ESCC at a younger age. The present findings strongly suggest that limiting the consumption of undiluted hard liquor may decrease the incidence of ESCC in the investigated population.

## Figures and Tables

**Table I:** Participant characteristics and association between individual risk factors and ESCC.

			Univariate conditional logistic regression	
			
Variable	Control no. (%)	Patient no. (%)	Unadjusted OR (95% CI)	P-value
Age^a^ (years)				
30–39	14 (2.6)	14 (2.6)		
40–49	135 (25.2)	135 (25.2)		
50–59	386 (72.2)	386 (72.2)		
Smoking and drinking status				
No smoking or drinking	179 (33.5)	119 (22.2)		
Smoking	185 (34.6)	150 (28.0)		
Drinking	37 (6.9)	38 (7.2)		
Both smoking and drinking	134 (25.0)	228 (42.6)		
Family history of esophageal cancer <0.001				
No	454 (84.9)	387 (72.3)	1.00 (Reference)	
Yes	81 (15.1)	148 (27.7)	2.10 (1.55–2.85)	
Combined smoking variable <0.001				
No smoking	216 (40.4)	157 (29.3)	1.00 (Reference)	
Amount^b^ ≤20/duration^c^ ≤20	146 (27.3)	138 (25.8)	1.25 (0.91–1.72)	0.171
Amount^b^ ≤20/duration^c^ >20	96 (17.9)	144 (26.9)	2.10 (1.49–2.95)	<0.001
Amount^b^ >20/duration^c^ ≤20	32 (6.0)	29 (5.4)	1.20 (0.69–2.08)	0.519
Amount^b^ >20/duration^c^ >20	45 (8.4)	67 (12.6)	2.04 (1.32–3.16)	0.001
Combined alcohol variable <0.001				
No drinking	364 (68.0)	269 (50.3)	1.00 (Reference)	
Amount^d^ ≤53.3/duration^c^ ≤20	92 (17.2)	89 (16.6)	1.26 (0.89–1.79)	0.196
Amount^d^ ≤53.3/duration^c^ >20	24 (4.5)	53 (9.9)	2.71 (1.62–4.54)	<0.001
Amount^d^ >53.3/duration^c^ ≤20	47 (8.8)	73 (13.6)	1.98 (1.32–2.95)	<0.001
Amount^d^ >53.3/duration^c^ >20	8 (1.5)	51 (9.6)	8.50 (3.81–18.94)	<0.001

ESCC, esophageal squamous cell carcinoma; OR, odds ratio; CI, confidence interval.

a Age used as a matching variable.

b Number of cigarettes/day.

c Duration (years) of alcohol intake or tobacco smoking.

d Amount of drinking (g ethanol/day).

**Table II:** Results of multivariate conditional logistic regression analysis and simulation study.

		Multivariate conditional logistic regression		
		
Risk factor	Age (years, means ± SD)	Adjusted OR (95% CI)	P-value	Simulation results [mean OR (95% CI)]
Family history of esophageal cancer (Reference = no esophageal cancer in relatives)			<0.001	
Yes	52±5.0	2.06 (1.48–2.85)^a^	<0.001	2.08 (1.49–2.90)^a^
Combined smoking variable (Reference = no smoking)			0.193	
Amount^b^ ≤20/duration^c^ ≤20	51±5.2	1.10 (0.77–1.56)	0.601	0.97 (0.69–1.37)
Amount^b^ ≤20/duration^c^ >20	50±3.3	1.58 (1.09–2.31)^a^	0.017	1.51 (1.02–2.22)^a^
Amount^b^ >20/duration^c^ ≤20	54±5.1	1.06 (0.59–1.90)	0.857	0.85 (0.48–1.52)
Amount^b^ >20/duration^c^ >20	53±3.3	1.30 (0.79–2.12)	0.302	1.33 (0.80–2.18)
Combined alcohol variable (Reference = no drinking)			<0.001	
Amount^d^ ≤53.3/duration^c^ ≤20	50±5.8	1.20 (0.83–1.74)	0.329	1.39 (0.97–2.01)
Amount^d^ ≤53.3/duration^c^ >20	54±3.4	2.28 (1.32–3.94)^a^	0.003	2.27 (1.30–3.95)^a^
Amount^d^ >53.3/duration^c^ ≤20	50±5.3	1.91 (1.25–2.92)^a^	0.003	2.01 (1.30–3.10)^a^
Amount^d^ >53.3/duration^c^ >20	53±3.4	7.25 (3.12–16.83)^a^	<0.001	6.44 (2.85–14.57)^a^

OR, odds ratio; CI, confidence interval.

a 95% CI did not include 1.

b Number of cigarettes/day.

c Duration (years) of alcohol intake or tobacco smoking.

d Amount of drinking (g ethanol/day).

## Abbreviations

CI: confidence interval
ESCC: esophageal squamous cell carcinoma
OR: odds ratio
